# Ensuring Best Practice in Genomic Education and Evaluation: A Program Logic Approach

**DOI:** 10.3389/fgene.2019.01057

**Published:** 2019-11-08

**Authors:** Amy Nisselle, Melissa Martyn, Helen Jordan, Nadia Kaunein, Alison McEwen, Chirag Patel, Bronwyn Terrill, Michelle Bishop, Sylvia Metcalfe, Clara Gaff

**Affiliations:** ^1^Australian Genomics Health Alliance, Melbourne, VIC, Australia; ^2^Genomics in Society, Murdoch Children’s Research Institute, Melbourne, VIC, Australia; ^3^Department of Paediatrics, The University of Melbourne, Melbourne, VIC, Australia; ^4^Melbourne Genomics Health Alliance, Melbourne, VIC, Australia; ^5^Melbourne School of Population and Global Health, The University of Melbourne, Melbourne, VIC, Australia; ^6^Graduate School of Health, University of Technology Sydney, Sydney, NSW, Australia; ^7^Genetic Health Queensland, Royal Brisbane and Women’s Hospital, Brisbane, QLD, Australia; ^8^Kinghorn Centre for Clinical Genomics, Garvan Institute of Medical Research, Sydney, NSW, Australia; ^9^St Vincent’s Clinical School, University of New South Wales Sydney, Sydney, NSW, Australia; ^10^Genomics Education Program, Health Education England, Birmingham, United Kingdom

**Keywords:** workforce, genomic medicine, program logic, theory, education, evaluation

## Abstract

Targeted genomic education and training of professionals have been identified as core components of strategies and implementation plans for the use of genomics in health care systems. Education needs to be effective and support the sustained and appropriate use of genomics in health care. Evaluation of education programs to identify effectiveness is challenging. Furthermore, those responsible for development and delivery are not necessarily trained in education and/or evaluation. Program logic models have been used to support the development and evaluation of education programs by articulating a logical explanation as to how a program intends to produce the desired outcomes. These are highly relevant to genomic education programs, but do not appear to have been widely used to date. To assist those developing and evaluating genomic education programs, and as a first step towards enabling identification of effective genomic education approaches, we developed a consensus program logic model for genomic education. We drew on existing literature and a co-design process with 24 international genomic education and evaluation experts to develop the model. The general applicability of the model to the development of programs was tested by program convenors across four diverse settings. Conveners reported on the utility and relevance of the logic model across development, delivery and evaluation. As a whole, their feedback suggests that the model is flexible and adaptive across university award programs, competency development and continuing professional development activities. We discuss this program logic model as a potential best practice mechanism for developing genomic education, and to support development of an evaluation framework and consistent standards to evaluate and report genomic education program outcomes and impacts.

## Introduction

Genomic medicine is rapidly being incorporated into routine healthcare (Manolio et al., 2015) and, due to advances in technology, demand will grow as the time and cost of genetic/genomic testing reduce (Stark et al., 2019b). There are longstanding concerns and evidence that health professionals not trained in genetics or genomics have rudimentary knowledge of these disciplines, and are neither equipped nor confident to adopt new genomic technologies into clinical care (Fuller et al., 2001; Carroll et al., 2011; Feero and Green, 2011; Houwink et al., 2011; Korf et al., 2014; Slade and Burton, 2016). The need for quality educational programs, activities, and resources (collectively referred to here as ‘education interventions’) to improve the knowledge of health professionals who are not trained in genomics is critical to the successful integration of genomics into routine healthcare (Carroll et al., 2011; Wildin et al., 2017).

We undertook a review of genomic education produced in Australia in 2016–17 (Janinski et al., 2018; McClaren et al., 2018) and found numerous genomic education interventions are developed and implemented across diverse contexts (for example, formal education or training versus continuing education), often in response to local healthcare system needs perceived by the educator. Interviews with program convenors (n = 32) revealed many interventions lacked clear learning objectives or evidence-based teaching and learning practices, and few convenors reported using needs assessments to inform programs or conducting evaluations of outcomes or development processes. Of the program convenors interviewed, only 13% had a tertiary qualification in education.

Program funders and stakeholders require evidence that education interventions have successfully met tangible outcomes (Gaff et al., 2007). If the pathway to achieving desired results is not clearly outlined prior to the implementation of an education intervention, it is difficult to deduce why, and how, the intervention produced the outcomes it did. Logic models delineate the key inputs, activities, and intended outcomes of programs. If presented with sufficient detail, program logic models can help to articulate a logical explanation as to how a program intends to produce the desired outcomes—its mechanism of action. Logic models can be used to describe whole programs, or parts of a program. For example, Horowitz and colleagues recently proposed the Genomic Medicine Integrative Research Framework as a “whole of system” logic model encompassing context, interventions, processes and outcomes to support those implementing genomic medicine (Horowitz et al., 2019), with genomic education defined as one type of intervention in their conceptual framework. Logic models not only provide an understanding of the reasoning underpinning a program, they can aid the planning of its evaluation (Horowitz et al., 2019).

**Figure 1:**
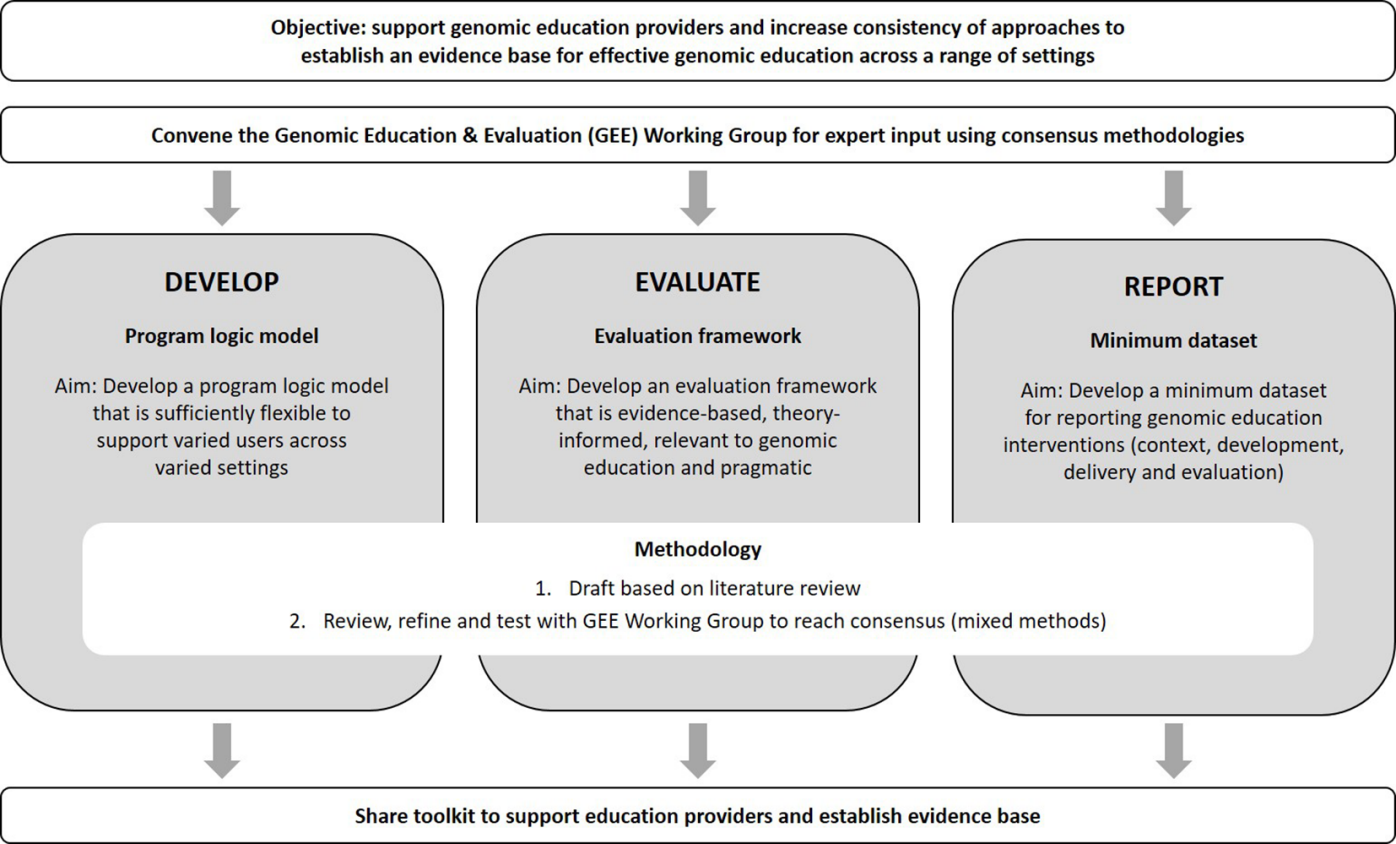
Summary of the Australian Genomics Workforce & Education “Effective Education” program of research.

Despite a need, there is little clarity on what defines quality genomic education interventions or successful strategies, and in which contexts (Wildin et al., 2017). Nor are there evidential standards around evaluating outcomes or reporting programs (Talwar et al., 2017). To begin to address this deficit, part of the Workforce & Education research program of the Australian Genomics Health Alliance (Australian Genomics; Stark et al., 2019a) aims to provide an evidence base for those developing genomic education (**Figure 1**). These include: 1) a program logic model to support design and development; 2) a framework for evaluation spanning the education lifecycle; and 3) a minimum dataset to report program design, development, delivery and evaluation.

Here we describe the consultative process of engaging education and evaluation experts in developing and testing a program logic model for genomic education. We also illustrate the logic model’s utility and flexibility across a variety of settings and contexts through narrative cases.

## Methods

### Context

The scope of “education” considered for this program logic is education for any professional, with or without specialized genetic training, regarding the application of genomic medicine. This spans clinical and laboratory professionals and, depending on the local context, clinicians may be primary, secondary or tertiary healthcare providers. For example, they may be family physicians/general practitioners who refer patients to genetic services or hospital-based medical specialists/physicians who refer patients or directly order genomic tests. Here we use the term ‘genetic specialists’ to denote people with specialized genetic training (clinical and/or laboratory) and ‘medical specialists’—including primary care physicians (PCPs)—as medically qualified individuals specialized in a sub-discipline other than genetics, who may refer or order genomic tests.

### Developing the Logic Model

A Working Group (SM, CG, AN, MM, and HJ) developed a draft program logic model from June through to December 2017. This was based on theories of program logic, evaluation, and adult learning principles and drew on the collective knowledge and experience in developing and applying program logic models to genetic education interventions and research.

The draft program logic was reviewed and refined in a 2-day co-design workshop involving 24 Australian and international genetic education and evaluation experts (see Acknowledgements) held in February 2018. All attendees were experienced in developing genetic or genomic education interventions, program evaluation and/or implementation science. The workshop included didactic, self-directed, and group activities to: develop a shared understanding of program logic structure and language; discuss the application of program logic and the associated evaluation framework to genetic and genomic education interventions; and review and refine the draft model. The workshop also considered the process for testing the logic model.

### Testing the Logic Model

To test the connections between the key elements of the logic model, clarify the intended outcomes and test feasibility, we applied a clarificative evaluation approach using authentic case studies (Owen and Rogers, 1999). A sample of workshop participants tested the draft model in local contexts, both Australian and in the UK. The model was subsequently applied to three genomic education interventions in the conception, planning or development stage, and retrospectively to a recently completed intervention. A template was used to capture data relevant to the development, delivery, and evaluation of the education intervention in each setting. The dataset included personal educator and institutional characteristics; the description of the intervention, including components of the logic model relevant to each setting, and applicability and usefulness of the model; evaluations planned and/or undertaken (type, evaluation questions, study design, findings, etc.); and documentation collected (collaboration agreements, project plans, meeting minutes, etc.). Draft narratives were verified by the participants and quotes were extracted from the dataset, email correspondence or notes made during conversations. These four participants also provided feedback on relevance and utility of the model to their setting.

## Results

### Overview of the Program Logic Model

**Figure 2:**
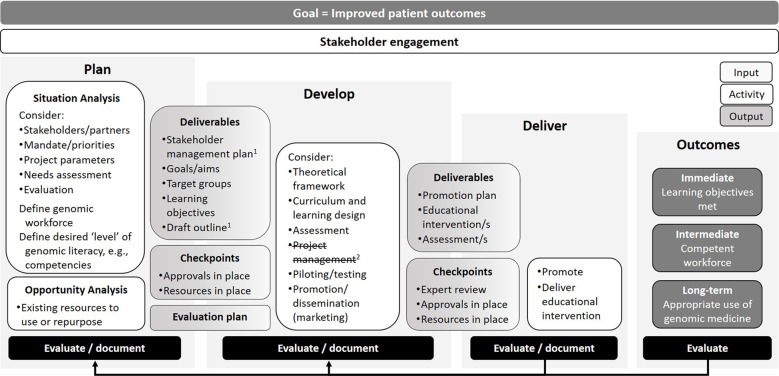
Program logic model for genomic education interventions. ^1^After testing the model in four contexts a stakeholder management plan was added as a Planning stage deliverable. ^2^Testing also clarified that Project management aspects can span all stages so this component was removed.

The logic model developed and refined by the working group and workshop participants captures four key components of the program cycle—planning, development, delivery, and outcomes—with goals, stakeholder engagement, and evaluation spanning all stages (**Figure 2**). Goals are the longer-term “ultimate” outcomes and, in the context of genomic education of health professionals, relate to improved patient outcomes. Stakeholders are people or organizations that are invested in the education intervention and evaluation. These can include funding agencies and sponsors, advocacy groups, learners, and those ultimately impacted by the intervention.

As the logic diagram depicts, the **planning** stage of the education intervention involves situation and opportunity analyses. A situation analysis considers numerous factors such as: stakeholders and partners, who may have mandates and competing priorities; project parameters (e.g., time, scope, budget); whether information exists, or can be gathered, around educational needs of target learners; and the target genomic workforce and level of genomic literacy. For example, depending on the context of the education provider (e.g., university lecturer versus clinician educator), if there is no current evidence on the genomic education needs of the target learner, the provider could conduct a needs assessment—if time and resources permit. At the minimum, this could involve assessing relevant stakeholder views on areas of genomic education that would better guide practice. An opportunity analysis may encompass potential partners (if not already identified), resources that can be repurposed or a literature review of, for example, competencies. The outputs of planning include a clearly-defined approach to stakeholder management (for example, frequency of meetings, reporting lines, etc.), goals, target groups, learning objectives, and a draft outline of the education intervention. Project management aspects overlap with components at all stages of the model. For example, at the end of the planning stage, it would be expected that education providers have all approvals and required resources in place. An evaluation plan should also be in place at this early stage—to foster transparency with stakeholders, identify questions, methods, and study design—before implementation and ensure sufficient resources for process and outcome evaluation are in place.

Activities in the **development** stage of the program logic model include evidence/theory-informed curriculum, content, and/or assessment design and development. There is growing evidence that education interventions that are based on clear theoretical foundations are more effective and have a greater impact on health professional educational outcomes than those without (Glanz et al., 2008; Bernstein, 2011). Adult learning theory is useful when considering strategies to cultivate the genomic medicine workforce, where skilled health professionals require continued education for immediate practical application (Gaff et al., 2007; Taylor and Hamdy, 2013). The development stage also includes activities related to project management to meet deadlines within budget and scope, then piloting the intervention (if appropriate) and also developing a promotion or marketing plan. The output of the development phase is an education intervention with a clear theoretical underpinning that has been planned, expertly reviewed and is ready to promote or market, with approvals and resources in place. Again, any decisions made during the development stage should be documented to allow later reflection and evaluation.

In the third stage, **delivery** of the education intervention, effective marketing is critical to success. Promotion is needed to ensure the target learners are aware of and use/attend/complete the education intervention. The education intervention is delivered or launched, including any assessment and/or immediate evaluation—such as pre-/post-workshop surveys or pop-up website user surveys—in addition to process documentation.

The fourth stage depicts the **immediate, intermediate, and long-term outcomes**. In the context of clinical genomic education, the immediate outcomes could relate to the learning objectives of the intervention, such as a change in knowledge, attitude, and skills. The intermediate outcome could relate to creating a competent genomic workforce, defined in relation to the aims of the education intervention (e.g., change in behavior). The long-term outcomes could include those related to the appropriate and timely use of genomic medicine, which then relates back to the overarching goal, which is improved patient outcomes. When constructing a program logic model to describe an education intervention, an education provider may use a series of “*if* … *then*…” statements (Owen and Rogers, 1999). For example, *if* learners complete this genomic education intervention and attain new skills (immediate outcome) *then* they become genomic-competent and practice accordingly (intermediate outcome), which *then* facilitates the appropriate use of genomic medicine (long-term outcome), which *then* improves patient care (ultimate goal).

**Evaluation** spans both the process of developing an education intervention and evaluating delivery (processes) and its impact—here defined as immediate, intermediate, and long-term outcomes.

### Testing the Program Logic Model

**Figure 3:**
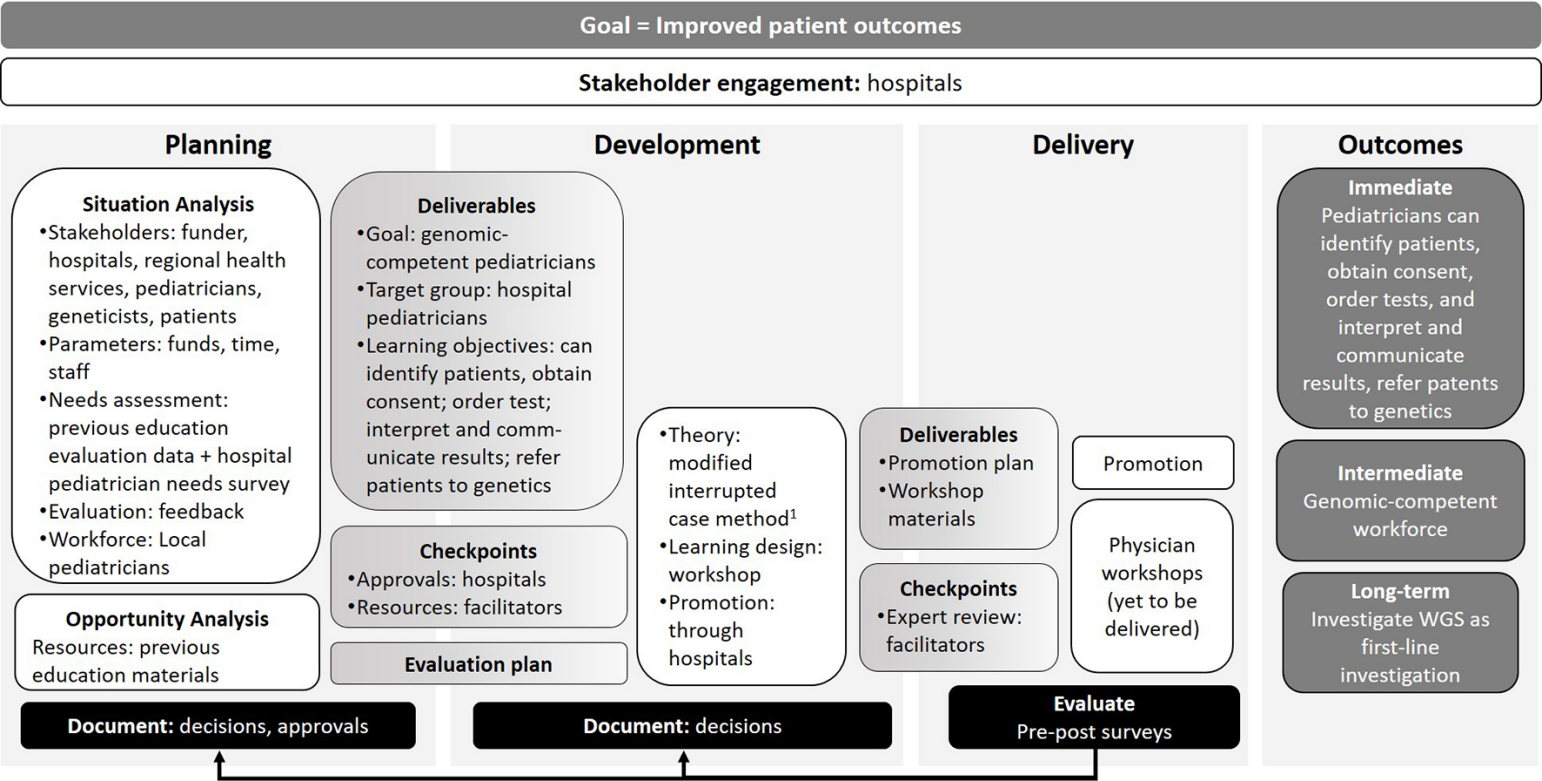
Illustration of how the different program logic model components map to the development of clinical genomic workshops for pediatricians. ^1^(Herreid, 2005).

**Figure 4:**
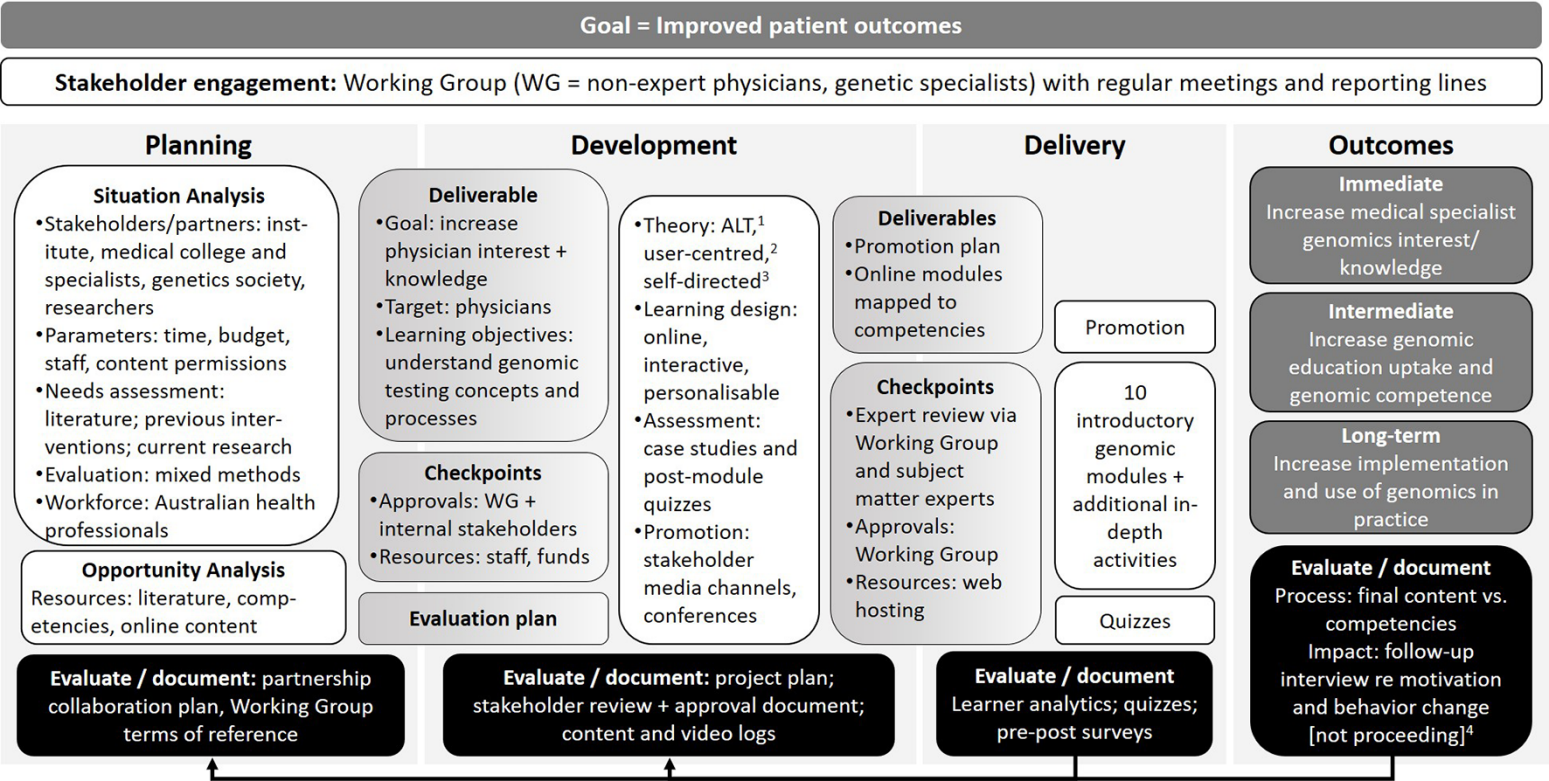
Illustration of how the program logic model can be used as a tool for reflection and targeted evaluation for quality improvement. ^1^Adult learning theory (Taylor and Hamdy, 2013); ^2^(Beetham and Sharpe, 2013); ^3^(Hase, 2009). ^4^The impact evaluation is not proceeding due to low participation.

Four workshop participants (CP, MB, BT, AM) tested the program logic model in local contexts. These included different countries (Australia and the UK) and different types of education intervention: workshops for pediatricians, a competency framework to support discussions around informed consent for genomic testing, online modules for medical specialists, and a university course. The four contexts involved varied outcomes, stakeholders and partners, and different organizations and resources. **Table 1** provides details of how each component of the program logic model was mapped to each context and the narratives below focus on different aspects of the model. Two narratives are described visually (CP and BT) in the program logic model format (**Figures 3** and **4**), with detailed program logic models for all four narratives included as **Supplementary Materials**. Use of the program logic model for each of the four contexts are described below, followed by the changes proposed and made to the program logic model following testing.

**Table 1:** Comparing components within the program logic model across four illustrative narratives.

Narrative	Using the model to plan workshops	Using the model for stakeholder management and reporting when developing competencies	Using the model for reflection and targeted evaluation for quality improvement	Using the model to support a cyclical co-design approach when developing a university course
**Person leading development of the intervention**	Clinician educator without education qualification	Clinician educator with education qualification	Science communicator with education qualification	Clinician educator with education qualification
**Goal**	Improve patient outcomes through improved healthcare services	Improve patient outcomes during consent for genomic testing as conversations are undertaken by competent health professionals	Improve patient outcomes as a result of improved physician understanding of, and interest in, genomic medicine	Improve patient outcomes through a genetic counseling workforce that is emergent and fit for purpose in the genomic era
**Stakeholder engagement**	Minimal, other than approvals	Multiple, clearly defined, extensive stakeholder engagement throughout with management and reporting plans, multiple boards and consultative events	Multiple, clearly defined, extensive stakeholder engagement throughout with regular meetings and reporting lines	Multiple, clearly defined, extensive stakeholder engagement throughout with regular meetings and consultations, plus a Curriculum Advisory Committee
**PLANNING**				
**Situation Analysis**				
Stakeholders/partners	Funder, hospitals, regional health services, pediatricians, geneticists, researchers, patients	Implementers of genomic medicine (all levels, including national health service, medical colleges, clinicians), patients	Institute, medical college, genetics society, physicians, researchers	University, professional society, genetic counselors (experienced and recent graduates), geneticists, medical specialists, ethicists, laboratory staff, indigenous health experts, learning designers, students, placement supervisors
Mandate/priorities	–	Health service mandates and priorities	College mandate (education) and priorities	University mandate (education plus research) and accreditation priorities
Project parameters	Budget, time, staff	Budget, time, staff	Budget, time, staff, content permissions	Budget, time, staff, research, accreditation and Australian Qualifications Framework^1^
Needs assessment	Previous education evaluation data; designed and deployed survey re hospital pediatrician needs Revealed need for workshops tailored to this group	Literature review Previous project evaluation data (consent materials; national analysis of individual learning needs) Revealed need for competencies for health professionals	Literature review Previous project evaluation data (genetic/genomic education interventions) Current local genomic workforce and education research Revealed need for introductory, short, accessible, online modules	Literature review Extensive stakeholder consultation Revealed need for blended learning course
Genomic workforce	Hospital-based pediatricians	Health professionals and education leads	Non-genetic health professionals	Genetic counselors
Desired level of genomic literacy	Become ‘comfortable’ with genomic medicine	N/A (developing competencies)	No current local competencies so undertook review and development of project-specific competencies; aim to become confident working with more experienced colleagues to order and act upon genomic tests	Mapped to local genetic counseling competencies
**Opportunity Analysis**				
Existing resources	Reviewed own previous education materials	Reviewed existing competencies	Reviewed existing online content	Reviewed existing online content
**Outputs/Deliverables**				
Goal	Genomic-competent pediatricians	Guidance for health professionals around consent for genomic testing	Increase medical specialist interest in, and knowledge of, genomic testing	Produce graduates of a new Master of Genetic Counseling who are fit to practice in the genomic era
Target group	Hospital pediatricians likely to be involved in the research program	English health professionals; education leads	Australasian non-genetic medical specialists	Genetic counseling students
Learning objective/s	Hospital-based pediatricians can identify patients, obtain consent; order test; interpret and communicate results, and refer patients to genetic services	N/A	Understand genomic testing concepts and processes	Course structure and subject-specific learning objectives
**Checkpoints**				
Approvals	Hospital	Board	Working group and internal stakeholders	Curriculum Advisory Committee and university academic board
Resources	None	Organization staff and resources	Institute staff	University staff, services and resources (learning design, library, marketing, student administration, etc.)
**Evaluation plan**	Pre-post quantitative study	Longitudinal mixed-methods study proposed	Longitudinal mixed-methods study proposed	Longitudinal mixed-methods study proposed
**DEVELOPMENT**				
Theoretical framework	Modified interrupted case method^2^	Competency-based CPD,^3^ reflective practice^4^ and self-directed learning^5^	Adult learning theory^6^ and user-centred,^7^ self-directed design^5^	Co-design principles^8^ and authentic learning^9^
Curriculum and learning design	Workshop presentations plus case content review by discipline-specific pediatricians	Consensus methodology used to develop competencies with stakeholders	Online, interactive, personalizable modules (informed by needs assessment)	Blended learning (mix of online and face-to-face learning)^10^ (informed by needs assessment)
Assessment	N/A	N/A	Case studies and post-module quizzes	Per subject
Piloting/testing	None	Iterative review through consensus methodology	Iterative review by Working Group	Iterative review by Curriculum Advisory Committee
Promotion or dissemination plan (marketing)	Through hospitals	Through medical colleges and stakeholders	Through stakeholder media channels and relevant medical professional conferences	Through university
**Outputs/Deliverables**				
Promotion plan	In place at this stage	In place at this stage	In place at this stage	In place at this stage
Educational intervention/s	Workshop content developed, including cases	Competencies developed	Online modules developed, aligned with stakeholder priorities	Subjects developed, aligned with accreditation requirements
Assessment/s	N/A	N/A	Additional in-depth activities + quizzes on organization website	Per subject
**Checkpoints**				
Expert review	By workshop facilitators	Iterative stakeholder review	Iterative stakeholder review Additional subject matter expert review when required Final content reviewed against competencies	Iterative stakeholder review
Approvals	N/A	Stakeholders; also seeking formal endorsement	Stakeholders	Curriculum Advisory Committee University and professional society accreditation
Resources	Clinical colleagues confirmed as workshop facilitators	Ongoing staff and resources	Ongoing institute staff Online modules hosted on college eLearning platform; additional resources hosted on organization website	Ongoing staff, services and resources, including lecturers and tutors employed specifically for the course
**DELIVERY**				
Promotion	To hospital staff	To medical colleges and on organizational website	To medical specialists through medical college, societies, conferences and social media	Advertised by university
Educational intervention	Workshops (yet to be delivered)	Competencies	10 online modules + additional in-depth activities	16 university subjects, including research and clinical placements First cohort of students (n = 24) enrolled in 2019
Assessment	N/A	N/A	Quizzes	Per subject
**OUTCOMES**				
Immediate	Hospital-based pediatricians can identify patients, obtain consent; order test; interpret and communicate results, and refer patients to genetic services	Awareness and use of competencies to identify learning needs	Increase physician interest in, and knowledge of, genomics	Launch course to meet genetic counseling profession needs, with sufficient enrolments to meet university requirements
Intermediate	Increase pediatricians’ comfort and competence with genomic medicine	Leaders and individuals use competencies to inform education and training, and inform development of future tools	Increase uptake of genomic education; increase medical specialists’ genomic competence by introducing concepts and processes of genomic medicine	Produce competent graduates who can practice genetic counseling in both genetic and genomic medicine settings
Long-term	Increase genomic literacy among hospital-based pediatricians who may be involved in a genomic medicine research program	Enable health professionals to know what is required to conduct conversations around genomic testing and facilitate informed patient decision-making	Increase use of genomics in practice; involved in broader genomic medicine integration	Develop, deliver, evaluate and refine a Master of Genetic Counseling that is future-focused, emergent, and fit for purpose in the genomic era
**EVALUATION**				
Process	Document decisions and approvals	Document decisions and approvals; effectiveness evaluation re promotion plan, access, adoption and use over time; review program evaluation	Document partnership collaboration plan, Working Group terms of reference; decisions and approvals, comparison of final content vs. competencies; content and video logs	Document decisions and approvals; post-subject and post-course student feedback (ongoing); staff and student reflections informing co-design approach (ongoing)
Impact	Pre-post surveys of changes in confidence and practice (yet to commence)	Change in individual/organizational competence (yet to commence)	Website learner analytics; quiz results; pre-post surveys of changes in interest and knowledge; follow-up interview re motivation and behavior change (not proceeding)	Long-term employer interviews (yet to commence)

^1^www.aqf.edu.au/aqf-levels; ^2^(Herreid, 2005); ^3^(Campbell et al., 2010); ^4^(Schon, 1983); ^5^(Hase, 2009); ^6^(Taylor and Hamdy, 2013); ^7^(Beetham and Sharpe, 2013); ^8^(McEwen et al., 2019); ^9^(Herrington and Oliver, 2000); ^10^(McGee and Reis, 2012).

#### Using the Model to Plan Workshops

Chirag is a medical geneticist with many years’ experience developing and delivering genomic education to health professionals. He has no formal education qualification, is not supported by a university or education organization and offers occasional genomic education interventions in addition to his usual clinical workload. Chirag recently obtained funding to lead a research program exploring the use of whole genome sequencing (WGS) as a first-line investigation for pediatric patients across several tertiary specialties. For this research program to become clinically embedded and successful in the long term, hospital-based pediatricians will need to know how to order and interpret WGS tests.

Chirag used the program logic model to plan and develop face-to-face workshops to increase genomic literacy among hospital-based pediatricians in his region (**Figure 3**). Chirag’s needs assessment (a survey of cross-disciplinary, tertiary hospital-based specialist pediatricians) revealed his target audience had limited genomic literacy and experience ordering and interpreting genomic tests. He used this information to help him define learning objectives and an evaluation plan to examine changes in confidence and practice. He obtained approval from the main pediatric tertiary hospital in his region to host the workshops, and a commitment to promote them to relevant staff. He also secured medical genetics colleagues to assist in teaching each workshop.

Chirag found the program logic model prompted him to include all the necessary considerations when planning genomic education for non-genetic health professionals.

*“It was great to have a formal document to use as a reference to consider all aspects of providing genomics education to non-genetics professionals. Many of the factors in the planning stage may not routinely be considered when planning smaller educational events (presentations to local departments), but clearly are essential in ensuring effectiveness and achievement of goals and outcomes for larger group educational activities like workshops. It allowed to me to ensure I had the correct resources and that I assessed the needs and current level of knowledge of my target audience, prior to designing the specific cases for the workshops.”*

#### Using the Model to Aid Stakeholder Management and Reporting When Developing Competencies

Michelle has a postgraduate qualification in genetic counseling, a PhD in genetic education, and several years’ international experience in genetic and genomic education. Michelle works for a national genomic education organization tasked with upskilling health professionals in genomic medicine. Previous work undertaken by Michelle’s organization identified a need for competencies to support education and training of health professionals who will undertake the consent conversation for genomic testing with patients or their family members.

Michelle used the logic model to assist stakeholder identification and to develop a stakeholder management plan for developing, disseminating and evaluating the competencies.

“*We established a Working Group to oversee the development of the competencies, and I used the key points outlined in the program logic to structure the Working Group discussions. I also use these points as a checklist to structure the progress reports I submit to our Assurance Board*.”

The initial stakeholder engagement activity included consultation with health service providers and laboratories, medical and nursing colleges and societies, and, at a separate event, consumer representative groups. All stakeholders agreed to the need for a set of competencies. The stakeholders wanted to *“outline the set of knowledge, skills and behaviors for ‘doing the job’ rather than what someone would achieve if they undertook a training session in this area”*.

Stakeholder consultation was also undertaken to develop a comprehensive mixed-methods evaluation plan that encompasses process and impact evaluation to both inform the refinement of the competence framework and answer the question: are health professionals who have genomic testing consent conversations competent in all areas of the competency framework? Michelle found the logic model helped stakeholders appreciate the importance of considering evaluation early in the planning and development phase.

“*Importantly, having the key points structured in a format that aligns to the resource development cycle means that key aspects such as defining the evaluation plan are considered throughout the development of the resource and not as an afterthought.*”

#### Using the Model for Reflection and Targeted Evaluation for Quality Improvement

Bronwyn has postgraduate qualifications in science communication and education, with many years’ experience planning, developing, delivering, and evaluating genetic education interventions. She works for a medical research institute, which has a genomics-focused education and outreach team that aims to improve Australasian health professionals’ understanding of, interest in, and use of genomics to facilitate its broader integration into healthcare. A previous needs assessment and opportunity analysis revealed a lack of short, accessible genomic education resources developed for Australasian medical specialists.

Planning for the educational intervention was already underway when Bronwyn participated in the program logic development workshop. Contributing to the development of the broader logic gave Bronwyn an opportunity to reflect on her own project, highlighting processes that she may have done differently or at a different stage (**Figure 4**). As current Australasian genomic competencies for medical specialists did not exist to benchmark the desired level of genomic literacy and guide stakeholder discussion on curriculum design, Bronwyn’s team reviewed existing international competencies^1^ to synthesize 66 competencies relevant to the project. The program logic model approach prompted Bronwyn and her team to map the final content against the agreed competencies, as multiple rounds of drafting and expert review during development had resulted in changes to the original outline. They found that 56 of the 66 competencies were covered in the modules, five were deliberately removed to reduce length and complexity, and five were unintentional omissions. The reflective process using the program logic model identified areas for improvement and, if resourcing allows, these omissions will be remediated.

In response to stakeholder input, the course was deliberately designed in a modular fashion.

“*A key decision at the development stage was to have an open learning pathway so the modules could be completed as whole, or learners could select sections most relevant to them*.”

However, learning analytics evaluation data reveal very few learners complete all modules. This has impacted the planned long-term evaluation of the modules, as recruitment information for the post-survey and 6-month follow-up interview was only included in the completion page of the modules, resulting in insufficient individuals being aware of the study.

Bronwyn reflected that the logic model is useful even for experienced educators:

“*As project planning was well underway by the time the program logic model was developed, it informed my input into the [international program logic model development] workshop. The aspects that extend across the model—stakeholder engagement and documentation/evaluation—are particularly valuable as reminders to review and assess the whole project at each stage. For example, a requirement to evaluate or document at both the planning and development stages may have meant we invested in a competency review at an earlier stage and/or broadened our stakeholder list.*”

#### Using the Model to Support a Cyclical Co-Design Approach When Developing a University Course

Alison is a clinician educator with an undergraduate education qualification and postgraduate genetic counseling and research qualifications. She is the Program Director for a new Master of Genetic Counseling course in Australia and used the program logic model to help monitor and manage a cyclical co-design process when developing, delivering and evaluating the course (McEwen et al., 2019). Alison’s university perceived a need for the new course driven by the growing demand for genetic counselors in Australia, mirrored internationally (Slade et al., 2015; Stewart et al., 2015; Hoskovec et al., 2018). Building on early stakeholder activities undertaken by the university to scope new allied health postgraduate degrees for development, the planning stage began with extensive stakeholder engagement activities (McEwen et al., 2018). These activities revealed, for example, that existing courses were oversubscribed for limited places, and taught primarily on-campus in only two major Australian cities. Offering a more accessible course—blended learning through synchronous and asynchronous interactive online activities, on-campus intensives and clinical placements—would assist with a stated aim of the program to increase the diversity of students entering the profession.

Alison used co-design principles throughout the course development process, supported by the program logic model. The program was three months into the 15-month planning and development phase when Alison attended the program logic development workshop. Alison found the logic model aligned well with the co-design process and informed the ongoing development and delivery of curricula.

“*The inclusion of frequent check points and evaluation activities is of particular importance/relevance to ensure the program is meeting the needs of the learners, and of the practicing genetic counselors who interact with them while on clinical placement.*”

The program logic provided Alison with a framework for an in-depth evaluation that goes beyond her university’s usual feedback processes. In addition to university-mandated student feedback surveys, students also provided evaluative feedback and staff completed a brief reflective survey for each subject, with the evaluations and feedback discussed at an ‘end of semester’ staff retreat. Survey data and feedback from class representatives further informed the co-design approach, with students providing ongoing feedback and suggestions to ensure the program is responsive to the experiences and insight of this core group of stakeholders.

Alison found the logic most helpful in illustrating the cyclical nature of planning, development, delivery and evaluation of the university award course, providing opportunities for ongoing improvement.

“*We use the program logic in a cyclic manner, to ensure we continue to reflect on the needs and goals of all the stakeholders involved, as we seek to deliver a robust and emergent genetic counselor education program.*”

### Refining the Model After Testing

Testing the model in local contexts revealed some tensions and areas for refinement. Three participants felt that ‘Project management’ components span all stages of the model, not just during the Development stage, as was shown in the draft version (**Figure 1**, footnote 2). Michelle noted, *“I’ve found the program logic incredibly helpful, not just to guide development of the competencies but also for the project management aspect, including reporting into our Delivery Board.”* While we acknowledge our logic model may be used as a project management tool, this was not the primary aim and the component was therefore removed from the Development stage.

The narratives highlighted the importance of identifying and engaging stakeholders and partners as early as possible, as these groups may influence decisions made in the planning stage. Based on feedback, a ‘Stakeholder management plan’ was added as an output to the Planning stage, along with a ‘Draft outline’, to help develop the evaluation plan, seek approvals and gather resources. As Bronwyn noted,

“*Identifying partnership opportunities early in the process, even as part of stakeholder analysis, allows you to leverage their expertise from the beginning, and helps ensure that their perspectives, requirements and constraints are incorporated into your plans…… Our partnership was established once there was already a project plan, timeline and budget in place. If the partnership was established earlier, we could have avoided updating the [draft] materials. So possibly identifying partners would be best mentioned in ‘Situation analysis’.*”

The feedback also confirmed the logic model can be used in a non-linear and/or iterative fashion. For example, Michelle and her team reviewed and refined the draft competencies though an iterative process using consensus methodologies with stakeholder representatives; health professionals from a range of disciplines then reviewed clinical scenarios at a workshop, mapping themes to the draft competencies and voting to highlight, and reduce, inconsistencies.^2^ These processes effectively combined the stage of Development with the component ‘Expert review’. After consideration, these were left as separate components of the model and potential overlap will be acknowledged in future companion documents.

Finally, additional tools were suggested during testing feedback that could further support genomic education development and evaluation. These included a list of organizations developing and evaluating genomic education interventions (to identify potential partners), a summary of the main education and evaluation theories, common learning designs and related assessments, expert review templates, and evaluation study designs. As Bronwyn noted, *“Even for experienced educators, I believe that a catalog of evaluation approaches and tools would be a valuable adjunct to this model.”*

## Discussion

To support education providers to plan, develop and deliver genomic education interventions that achieve their goals and meet stakeholder needs, we have developed a ‘generic’ program logic model as part of a toolkit to support effective development, evaluation and reporting of genomic education. To optimize the model’s relevance and usefulness, we used a structured, mixed-methods approach to develop a draft model, combining a literature review, expert input *via* iterative workshop activities to achieve consensus, then clarificative evaluation in local contexts to test the stages and activities within the program logic model against our aim (Owen and Rogers, 1999). The four narratives illustrated how the model can be helpful to a range of education providers (with or without education qualifications) across a range of contexts, spanning smaller, more *ad hoc* interventions to larger, clearly-structured, mandated and well-funded interventions. While the model was not designed as a project management tool, several workshop participants were also project managers, so these aspects may have permeated the draft model as a result. Many people who develop and/or provide genomic education interventions may also be project managers and may use this model in a different way to someone who is using it to, for example, inform a theoretical framework.

The program logic developed in this paper is a versatile and useful tool for developing education interventions in different settings. Despite a “call to action” over a decade ago (Gaff et al., 2007), few papers published since have described use of program logic in their design or evaluation. This program logic model can be used to inform program development and redesign; it is not intended to be linear, but as with all program logic models, can be used through cycles, with the outputs and outcomes informing inputs and activities at different stages. As not all education providers will be familiar with a program logic model approach to developing interventions, we are developing a set of companion documents to support the use of the tool, including a “how to” guide, a glossary of terms, useful resources for both education and evaluation, and detailed definitions and examples throughout.

This model was developed with input from members of the Genomic Education and Evaluation Working Party (see Acknowledgements). These included education developers and providers from independent and government-funded organizations (e.g., Centre for Genetics Education, NSW Health; Health Education England) as well as research institutes (e.g., The Jackson Laboratory and Garvan Institute of Medical Research) and universities (e.g., University of Ottawa, University of Melbourne). While some members may have had past industry experience, none were able to provide current industry perspectives. The model may therefore be further strengthened by testing in industry and other contexts. Similarly, while deficits in the draft model were identified and addressed by expert consensus during development and testing, we expect that this will be the start of an iterative process as others use the model; we therefore encourage those who use the model to contact us to provide feedback.

Program funders typically require evidence of achieving genomic education intervention aims and objectives however, it consistently proves difficult to gather robust evaluation data for genetic education interventions, with even simple utilization statistics sometimes difficult to ascertain (Wildin et al., 2017). The program logic is a tool to support development of genomic education interventions; now the challenge is to evaluate these interventions using consistent approaches that reflect best practice in evaluation. This will also help to build an evidence base of “quality genomic education,” to begin to define outcomes and impacts across different settings (Khoury et al., 2009; Wildin et al., 2017). These endeavors may be assisted by an evaluation framework for genomic education and standards for the description of genomic education interventions and evaluation outcomes.

Our proposed suite of tools to develop, evaluate and report genomic education interventions will enable education providers and researchers to begin to establish an evidence base of effective genomic education and evaluation practice. We are currently using the model to develop, deliver and evaluate continuing genomic education interventions from the ground up. Over time, we expect that other education providers will provide feedback on use of our program logic model in many different contexts. This relies on effective dissemination of iterations of the tool: effectively promoting and sharing tools and resources is a challenge generally. Reviews of genetic and genomic education interventions (see for example, (Haga, 2006; Talwar et al., 2017) quickly become outdated and are sporadic. Repositories created by specialist colleges or organizations may be helpful within disciplines but require funding for sustainability and maintenance and may be hidden behind membership firewalls, reducing accessibility. There are many high-quality genomic education repositories^3^ but to the best of our knowledge there are no open-access repositories for genomic education development, evaluation and reporting with international examples. For example, a repository of program logic models describing genomic education interventions could be useful in showing how, over time, interventions change and adapt, and the reasons for these changes. This is the focus of ongoing research in our group but it is challenging to find. We have created a local network of genomic education and evaluation professionals (the Genomic Education Network of Australasia) to share research findings and exemplars of education and evaluation tools and networks will also be used for disseminating internationally. We recognize that sustainable, long-term hosting and dissemination of this model and body of work is necessary and continue to explore appropriate local and international options. Establishing and incorporating this evidence base is critical in the development of effective genomic education interventions that can be tailored to the needs of the audience.

## Data Availability Statement

All datasets generated for this study are included in the **Supplementary Files**.

## Author Contributions

CG and SM conceived the idea for the publication. AN, CG, SM, MM, HJ, AM, CP, BT and MB had intellectual input into the program logic model and preparation of the manuscript. NK provided intellectual input into preparation of the manuscript. All authors approved the final version and agree to be accountable for all aspects of the work.

## Funding

This work was supported by the Victorian Government’s Operational Infrastructure Support Program and a grant from the Australian National Health & Medical Research Council (GNT1113531).

## Conflict of Interest

CG and SM, are co-editors of the special issue where this manuscript is featured; AN and MM are affiliated with CG and SM. CG and SM were not involved in the editorial or peer-review process. The remaining authors declare that the research was conducted in the absence of any commercial or financial relationships that could be construed as a potential conflict of interest.

## Footnotes

1 Inter-Society Coordinating Committee for Physician Education in Genomics (ISCC) Physician competencies; National Coalition for Health Professional Education in Genetics: Core Competencies for all Health Professionals added to by Callier et.al; European Society of Human Genetics: Core Competences in Genetics for Health Professionals in Europe.

2 www.genomicseducation.hee.nhs.uk/images/pdf/Meeting%20for%20Consent%20Competency%20Framework%2012-02-2019.pdf

3 See, for example, Health Education England&amp;rsquo;s Genomics Education Programme (www.genomicseducation.hee.nhs.uk/), Genetics Education Canada – Knowledge Organization (https://geneticseducation.ca/), the Genetics/Genomics Competency Centre (G2G2; https://genomicseducation.net) or The Jackson Laboratory (www.jax.org/education-and-learning).
