# Supplementary material for: Ensuring Best Practice in Genomic Education and Evaluation: A Program Logic Approach
Source: Front Genet. 2019 Nov 8;10:1057. doi: 10.3389/fgene.2019.01057 (PMC6857516; doi:10.3389/fgene.2019.01057)
Supplement: Supplementary file 1 [file DataSheet_1.pdf]

Ensuring best practice in genomic education and evaluation: a program logic approach

Nisselle A, Martyn M, Jordan H, Kaunein N, McEwen A, Patel C, Terrill B, Bishop M, Metcalfe S & C Gaff on behalf of the Genomic Education and Evaluation Working Party

Frontiers in Genetics, 2019

Supplementary Materials

Detailed program logic models describing all four local contexts:

Supplementary Figure 1. Mapping the different program logic model components to the development of clinical genomic workshops for pediatricians.

Supplementary Figure 2. Using the different program logic model components for stakeholder management and reporting when developing competencies.

Supplementary Figure 3. Using the program logic model as a tool for reflection and targeted evaluation for quality improvement.

Supplementary Figure 4. Mapping the different program logic model components to a cyclical co-design approach when developing a university course.

Supplementary Figure 1. Mapping the different program logic model components to the development of clinical genomic workshops for pediatricians.

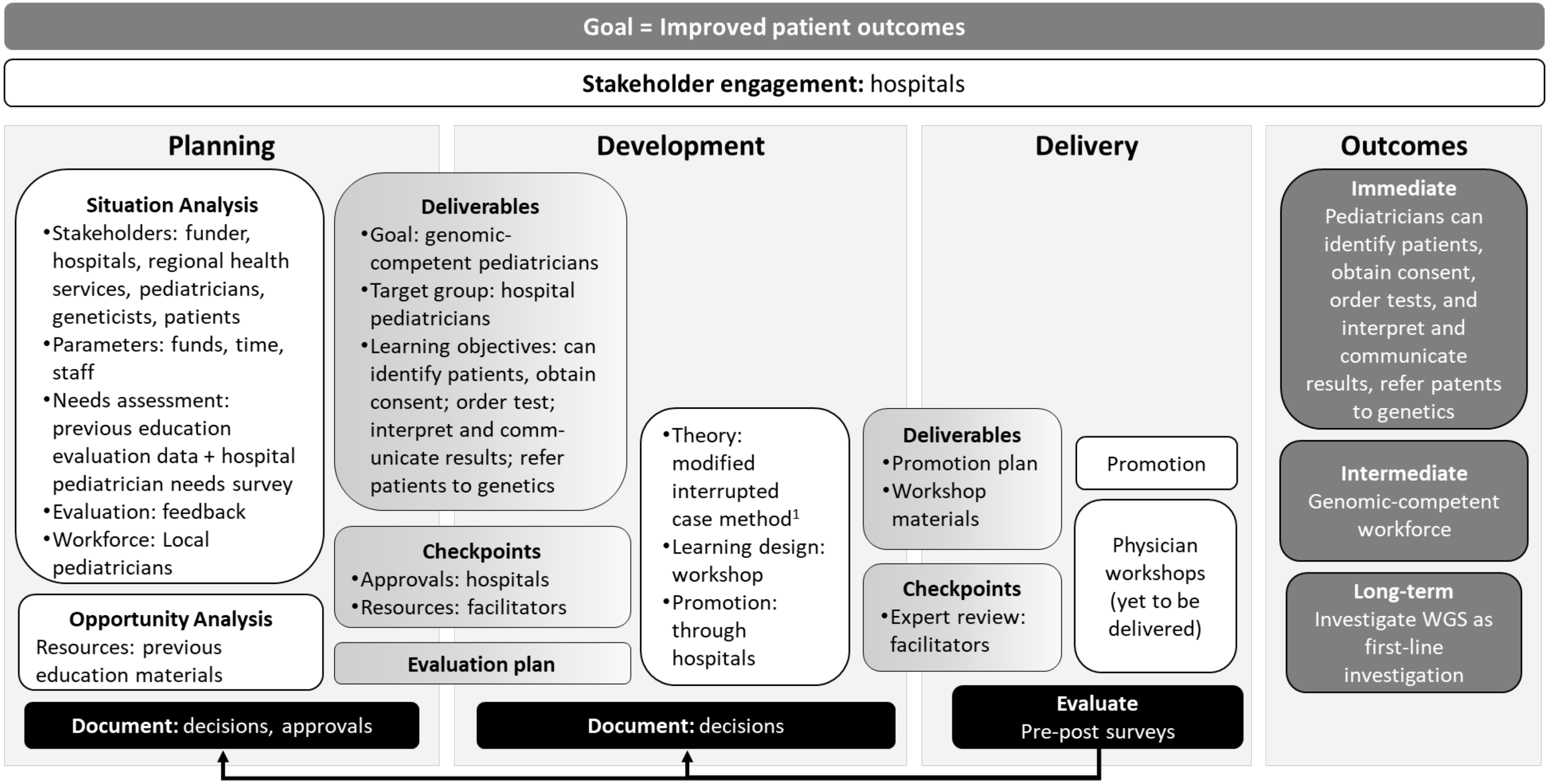

[1] (Herreid, 2005).

Supplementary Figure 2. Using the different program logic model components for stakeholder management and reporting when developing competencies.

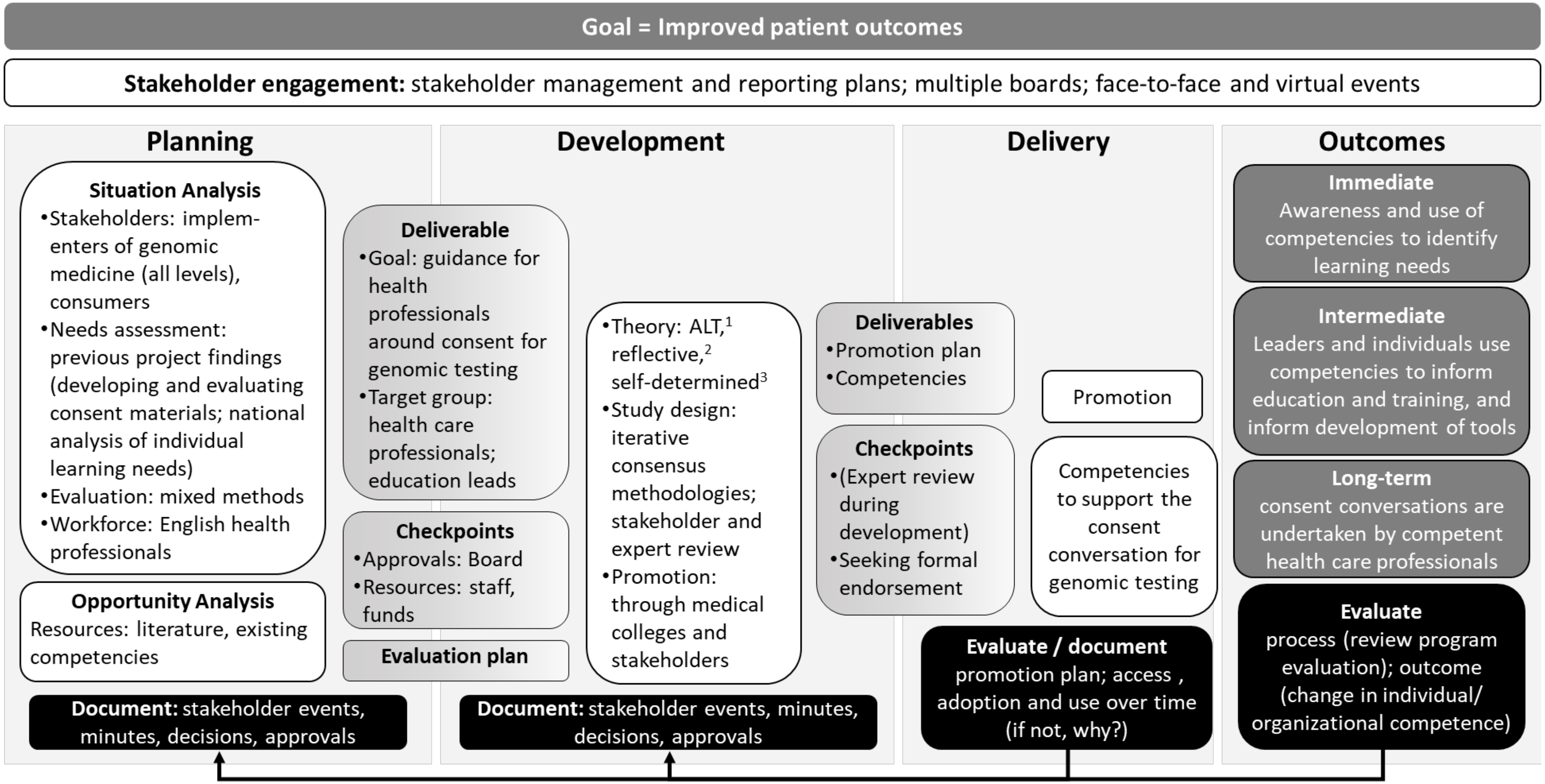

[1] Adult learning theory (Campbell et al., 2010); [2] (Schon, 1983); [3] (Hase, 2009).

Supplementary Figure 3. Using the program logic model as a tool for claricative evaluation during development of online modules for medical specialists.

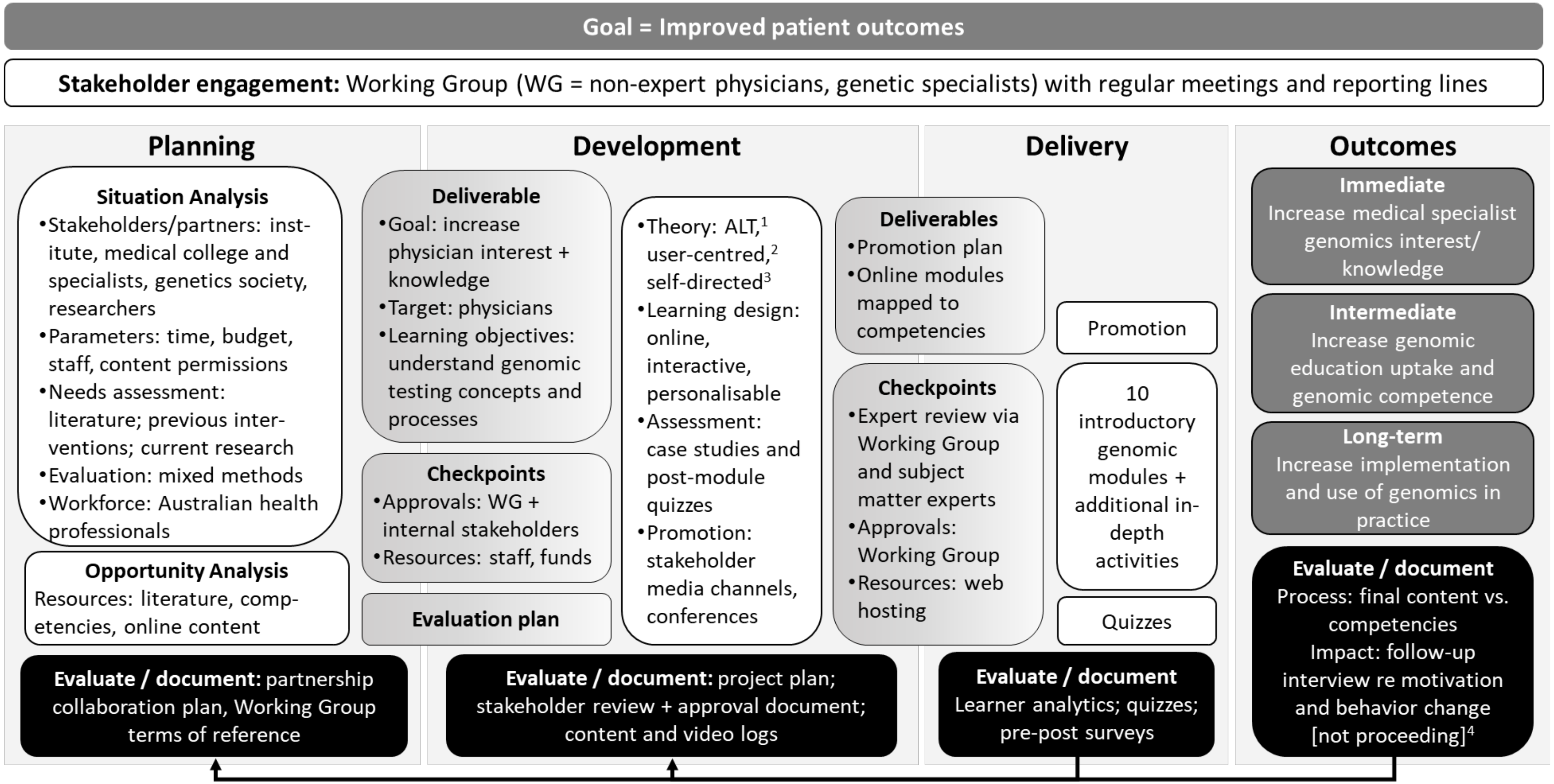

[1] Adult learning theory (Taylor and Hamdy, 2013); [2] (Beetham and Sharpe, 2013); [3] (Hase, 2009).

Supplementary Figure 4. Mapping the different program logic model components to a cyclical co-design approach when developing a university course.

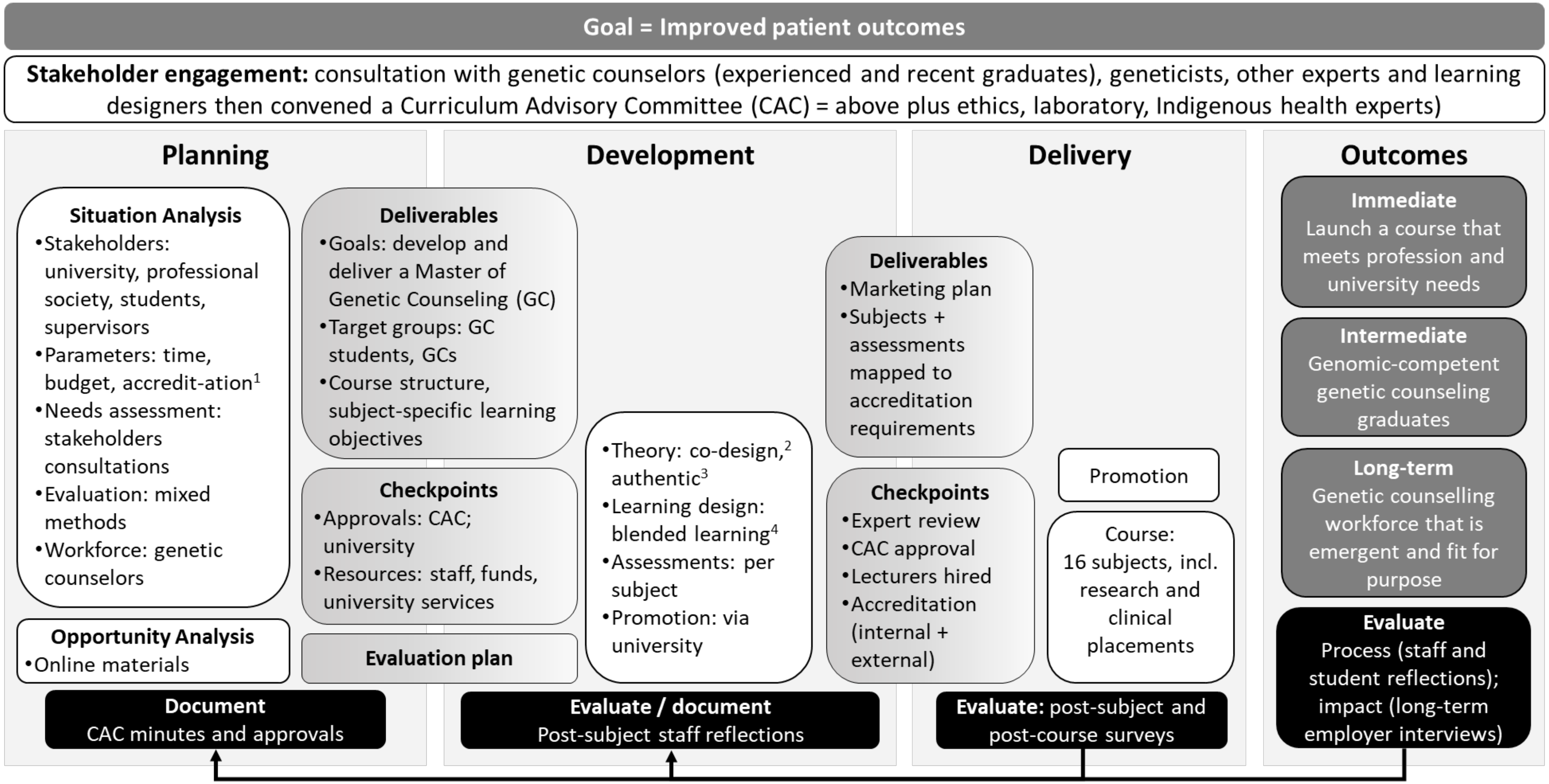

[1] including the Australian Qualifications Framework (www.aqf.edu.au/aqf-levels); [2] (McEwen et al., 2019); [3] (Herrington and Oliver, 2000); [4] (McGee and Reis, 2012).
